# Telemedicine in Neonatal Home Care: Identifying Parental Needs Through Participatory Design

**DOI:** 10.2196/resprot.5467

**Published:** 2016-07-08

**Authors:** Kristina Garne, Anne Brødsgaard, Gitte Zachariassen, Jane Clemensen

**Affiliations:** ^1^Hans Christian Andersen Children's HospitalOdense University HospitalOdenseDenmark; ^2^Paediatric DepartmentCopenhagen University Hospital HvidovreHvidovreDenmark; ^3^Centre for Innovative Medical TechnologyOdense University HospitalOdenseDenmark

**Keywords:** preterm infant, telemedicine, participatory design, neonatal home care

## Abstract

**Background:**

For the majority of preterm infants, the last weeks of hospital admission mainly concerns tube feeding and establishment of breastfeeding. Neonatal home care (NH) was developed to allow infants to remain at home for tube feeding and establishment of breastfeeding with regular home visits from neonatal nurses. For hospitals covering large regions, home visits may be challenging, time consuming, and expensive and alternative approaches must be explored.

**Objective:**

To identify parental needs when wanting to provide neonatal home care supported by telemedicine.

**Methods:**

The study used participatory design and qualitative methods. Data were collected from observational studies, individual interviews, and focus group interviews. Two neonatal units participated. One unit was experienced in providing neonatal home care with home visits, and the other planned to offer neonatal home care with telemedicine support. A total of 9 parents with preterm infants assigned to a neonatal home care program and 10 parents with preterm infants admitted to a neonatal unit participated in individual interviews and focus group interviews, respectively.

**Results:**

Three overall themes were identified: being a family, parent self-efficacy, and nurse-provided security. Parents expressed desire for the following: (1) a telemedicine device to serve as a “bell cord” to the neonatal unit, giving 24-hour access to nurses, (2) video-conferencing to provide security at home, (3) timely written email communication with the neonatal unit, and (4) an online knowledge base on preterm infant care, breastfeeding, and nutrition.

**Conclusions:**

Our findings highlight the importance of neonatal home care. NH provides parents with a feeling of being a family, supports their self-efficacy, and gives them a feeling of security when combined with nursing guidance. Parents did not request hands-on support for infant care, but instead expressed a need for communication and guidance, which could be met using telemedicine.

## Introduction

### Overview

Birth below 37 weeks of pregnancy requires admission to a neonatal intensive care unit (NICU), which can have long-term impacts on the family. A growing body of literature recognizes the importance of family-centered care (FCC) in the NICU [1]. However, having an infant in the NICU is still stressful because mothers have difficulty bonding with their infants and have feelings of not being a parent [2-3]; also, parents must divide their time between the NICU and home [4], especially when there are other children in the family [5]. For the majority of preterm infants, the last weeks in the NICU involve tube feeding and establishment of breastfeeding [4-5]. Establishment of breastfeeding is challenging, lasts several weeks, and cannot be accelerated because it depends on infant maturity [4-5]. Neonatal home care (NH) was developed to meet FCC requirements and optimize family conditions at this time [4,6-9]. NH allows infants to remain at home for tube feeding and establishment of breastfeeding [9]; this occurs with regular home visits from neonatal nurses until the infant can eat and gain weight without supplemental tube feeding, meeting criteria for discharge. NH seems to increase breastfeeding rates [8,10], increase parent self-confidence, support early family formation [11], and provide the benefits of FCC [9]. Thus, NH can provide a desirable transition from the NICU to home for preterm infants and their parents. However, for hospitals covering large regions, home visits may be challenging, time consuming, and expensive and alternative approaches must be explored. In the current health care environment, the need to reduce overall costs despite increasing admissions has led to treatments moving from the hospital to the home, and telemedicine provides a further opportunity to reduce costs [12]. Decreasing costs in the neonatal field is crucial [13-14] and early discharge is one approach. This study was the first to investigate whether telemedicine could provide a viable alternative to home visits. Despite little research on telemedicine in the neonatal field [15], one study has shown telemedicine to offer a communication platform between the NICU and the home and to provide parental satisfaction [6].

### Aim

The aim of this study was to identify parental needs when preterm infants receive NH with telemedicine support.

## Methods

### Design

User involvement in health care research is important [16], especially when developing new health technologies, such as telemedicine. Such involvement is imperative to ensure that these technologies meet the needs of the users [17-20]. This study employed a participatory design (PD) approach. PD is typically carried out in three phases [17-18]; this article reports on interviews and focus group interviews from Phase 1. The next steps will be to develop a telemedicine solution (Phase 2), addressing the needs identified in this study, and to implement that solution (Phase 3), which will be the topic of future papers.

### Setting and Participants

Parents with preterm infants admitted to two NICUs (see Table 1)—Hvidovre Hospital (HH) and Hans Christian Andersen Children’s Hospital (HCA)—participated in this study (see Table 2). HH has experience in providing NH with home visits and HCA planned to offer NH with telemedicine support.

**Table 1:** Characteristics of neonatal intensive care units involved in the study.

Characteristics	Hvidovre Hospital	Hans Christian Andersen Children’s Hospital
Associated hospital	Copenhagen University Hospital	Odense University Hospital
Location	Capital region	Region of Southern Denmark
Type of unit	Neonatal intensive care unit	Neonatal intensive care unit
Neonatal home care experience	Experienced in providing neonatal home care with home visits	Planning to offer neonatal home care with telemedicine

### Data Collection

Observational studies, individual interviews, and focus group interviews were conducted. The observational studies monitored the dynamic and actions during NH home visits, following recommendations from Spradley [21]. Based on the observational study, a semistructured interview guide inspired by Malterud [22] and Kvale [23] was developed to interview parents of preterm infants assigned to NH from the NICU at HH. These interviews aimed to identify parent experiences with NH and home visits, including the meeting content and meaning. The four themes of the interviews were as follows: home visits, parent self-efficacy, nutrition/breastfeeding, and family life. From December 2014 through January 2015, the first author (KG) interviewed parents in their homes while their preterm infants received NH.

Parallel to the interviews, two focus group interviews were conducted with parents whose preterm infants were admitted to the NICU at HCA who fulfilled the inclusion criteria for NH, which were as follows: (1) stable infant without need to monitor vital signs, (2) minimum age equivalent of 34 weeks, and (3) tube feeding required. Both primiparous and multiparous parents were included in the focus group interviews. The focus group interviews aimed to identify norms, ideas, and practices [22]. Themes were as follows: the last weeks in the unit, nutrition/breastfeeding, and needs when receiving NH supported by telemedicine.

The first (KG) and fourth (JC) authors led the focus group interviews. Field notes were taken by the fourth author (JC) during the interviews. All interviews were recorded and fully transcribed by the first author (KG). A total of 9 parents participated in an individual interview, and 10 parents participated in focus group interviews (see Table 2). Interviews lasted 34-70 minutes, and focus group interviews lasted 86-99 minutes.

### Analysis

Interview data were analyzed using Malterud’s systematic text condensation [24], which consists of four steps (see Table 3) as follows: (1) read transcripts repeatedly to identify themes, (2) identify and code units of meaning, (3) identify subgroups of codes from step 2 and develop condensates from them, and (4) describe experiences based on the condensates [24]. To have multiple perspectives during the first process of the analysis, the first (KG) and second (AB) authors individually extracted themes from the individual interview and focus group interview transcripts. These themes were triangulated and a consensus was reached. The first author (KG) completed steps 2-4, and the final product was approved by all authors.

**Table 2:** Demographic data for parents and infants (n=19).

Demographic information		Hvidovre Hospital (n=9)	Hans Christian Andersen Children’s Hospital (n=10)
Mothers, n (%)		5 (56)	7 (78)
Mothers’ age in years, range		25-41	24-36
First-time mothers, n (%)		3 (33)	4 (44)
**Mothers’ education, n (%)**			
	Student	0 (0)	1 (11)
	10-13 years	2 (22)	2 (22)
	>13 years	3 (33)	4 (44)
Fathers, n (%)		4 (44)	3 (33)
Fathers’ age in years, range		28-40	26-36
First-time fathers, n (%)		2 (22)	3 (33)
**Fathers’ education, n (%)**			
	Student	0 (0)	2 (22)
	10-13 years	2 (22)	0 (0)
	>13 years	2 (22)	1 (11)
**Infants' information, range**			
	Gestational age(weeks+days)	28+5-34+4	30+4-35+3
	Birth weight (grams)	1100-2375	875-2550

**Table 3:** Example of the analytical process^a^.

Setting and initial themes		From themes to codes		Subcategories	Overall category
		Quotes	Codes		
**Hans Christian Andersen Children’s Hospital**					
	Family conditions	“Out here you are a part-time family and that’s the way it will be” (Father #5).	Families were separated during their infant’s admission and it affected their perception of being a new family.	Separation of families	Being a family
	Being away from home	“It lasted for so long [breastfeeding establishment], because everything went so slowly. We had been here for so long that we could do all the things the nurses did, so you felt you wasted your time and would have benefitted from being home” (Mother # 6).	Families felt staying in the neonatal unit during breastfeeding establishment was a waste of time and that made them long for home.	Longing for home	Being a family
**Hvidovre Hospital**					
	Paternal role	“When I got there [to the NICU^b^] the time was so short that is was a lot of work for a one-hour visit. I’m now a dad rather than being a helper. Definitely“ (Father #1).	Fathers experienced that NH^c^ gave them possibilities of fathering their infants.	Room for fatherhood	Being a family
	Daily life	“Obviously, we can do our own things; we are not just sitting in the hospital. Now we can carry on, do what we want and get on with our lives” (Father).	Families experienced a return to their daily life. Further NH brings calmness to the family being at home instead of in the hospital. They feel it is better to be home.	Own surroundings brings comfort	Being a family

^a^The four-step analytical process [24]: (1) withdrawing themes, (2) identifying meaning of units and coding them, (3) identifying different subgroups, and (4) description of experiences based on the subcategories.

^b^NICU: neonatal intensive care unit.

^c^NH: neonatal home care.

### Ethics

According to the Declaration of Helsinki, respondents received written and oral information about the study and provided signed consent. They could withdraw consent at any time with no consequences for future treatment of themselves or their infants. The study was approved by the Danish Data Protection Agency (2008-58-0035). The study was presented to the local ethics committee, but their review was deemed to not be required by Danish law.

## Results

Analysis of the individual interviews and focus group interviews identified the following three categories: being a family, parent self-efficacy, and nurse-provided security.

### Being a Family

Parents expressed that the admission of their preterm infant to the NICU negatively affected being a new family, because they felt separated; only mothers were allowed to stay 24 hours per day with their infant. As one father stated, “Out here you are a part-time family and that’s the way it will be.” Many mothers were frustrated that their husbands could not spend the night, because they needed their support and wanted to share the experience.

Parents felt that staying in the neonatal unit with a healthy preterm infant for tube feeding and establishment of breastfeeding was not necessary:

It [establishment of breastfeeding] lasted for so long because everything went so slowly. We had been here for so long that we could do all the things the nurses did, so you felt you wasted your time and would have benefitted from being home.

Mother #6

Finally, parents felt trapped by the physical environment of the neonatal unit where strict hygiene rules required them to ask nurses for diapers, washcloths, and other basics required for infant care. This gave them a feeling of dependency on the nurses, which they disliked.

Parents with a preterm infant assigned to NH expressed that the NH supported their perception of being a family. There were overall positive effects of NH, including a feeling of calm for the entire family. One father stated the following:

Obviously, we can do our own things; we are not just sitting in the hospital. Now we can carry on, do what we want and get on with our lives.

Father #1

Parents were relieved to be in charge and not to have to adapt to hospital rules. For families with other children, NH allowed them to get back to being a normal family. For men, NH provided the opportunity to father their infants, becoming more familiar with their noises and cries, which they did not observe while their infants were in the hospital. One father stated the following:

I had no time with my daughter [in the NICU] because when I got there, the time was so short that it was a lot of work for a one-hour visit. I’m now a dad rather than being a helper.

### Parent Self-Efficacy

Parents with infants in the NICU felt that the constant presence of nurses negatively affected their decision making. When nurses entered the room to help the parents, it made parents doubt their competence instead of supporting their self-efficacy. One mother stated the following:

Today, for example, when they [twins] needed a bath, a nurse came in and said, “Shouldn’t I help you?” It makes me insecure. Can’t I do that myself?

Parents receiving NH experienced an increase in their self-efficacy by being at home and parent-infant bonding was further strengthened:

Being home means that you bond with your child because when you are in the hospital among professionals, you get the feeling that they are more capable of caring for him, and that others are better at taking care of your child is a terrible feeling. Here at home, we are absolutely the best at caring for him, which gives us a totally different feeling.

Mother #2

### Nurse-Provided Security

Parents with infants in the NICU were happy with guidance from nurses when establishment of breastfeeding was prolonged. One mother stated the following:

There has been someone [a nurse] that has guided me all the way and said that it was okay, that breastfeeding establishment takes time.

Mother #2

Nurses also provided parents with a reassurance of normality:

I think it was important to know. Some kind of normality in such an abnormal situation.

Mother #1

Similarly, for parents of infants receiving NH, home visits from neonatal nurses gave them a feeling of security that the infant was doing fine:

If she didn’t come, then I might look at him and say doesn’t his belly looks a little dilated? The way that a professional sees him and says he is doing fine, is so fantastic.

Mother #3

Frequent professional eyes on the infants reassured parents that their infant was developing normally. For this reason, the parents suggested the use of a telemedicine device to serve as a “bell cord,” giving 24-hour access to the neonatal unit. One father stated the following:

A video connection would be good if we needed the nurse to see some red spots on the baby or help with the tube, but it could also be by email, if you had a question for which you didn’t need the answer right away, but just within a couple of hours.

Father #3

Timely email communication with the neonatal unit gives parents the opportunity to communicate when needed. This, combined with an online knowledge base regarding preterm infant care, especially breastfeeding and nutrition, would provide security and self-efficacy.

## Discussion

### Principal Findings

This study supports the use of NH because it gives parents the feeling of being a family and promotes their self-efficacy. NH provides parents with the guidance they need from the nurses when they do not require hands-on support.

Studies have demonstrated the importance of FCC when preterm infants are admitted to the NICU, showing positive effects on parent-infant relationships and parents' experience of being involved in infant care [1,25]. Despite the NICU at HCA performing FCC, parents experienced a lack of involvement and questioned their own decision-making regarding infant care; the hospital environment and presence of health care professionals affected parents’ thinking and actions. It has been shown that the NICU milieu can be a barrier for parent-infant bonding [26], contrary to NH [9-11]. Statements from parents with an infant receiving NH support the findings from Turner et al [26]: they experienced growing independence, increased decision-making, and a feeling of the infants as their children. When parents are successful in decision-making, it increases their self-efficacy [27] and they experience increased empowerment. The noisy, busy environment of the NICU is a barrier for establishment of breastfeeding [26] and is stressful for parents. Studies suggest that establishment of breastfeeding might benefit from NH [8-10], and parental responsibility for infant feeding and general care reduces parental stress [28].

Our findings revealed that nurse guidance of infant care, rather than hands-on support, provided a feeling of security. This supports telemedicine as an alternative to hospital admission. Gund found that video-conferencing could reduce the need for home visits and was less stressful than home visits [6]. However, Lindberg found that video-conferencing could not replace ordinary care [29]. This inconsistency may be explained by the 4-year time difference between these two studies, during which our adaption to technology has increased rapidly. The methods used in these prior studies are not in accordance with recommendations for implementing telemedicine in clinical practice today [30-31], since the users’ needs were not identified before using telemedicine. Identifying needs of users before developing a telemedicine device can reduce the risk of implementation failure in clinical practice [31]. However, the findings in this study were in accordance with recommendations, and the development of a telemedicine device covering all parental needs is feasible.

### Study Limitations

This study had a small sample size. Infant gestational age and birth weight varied, and the parents represented a heterogeneous group varying in age, education, and primiparous versus multiparous status. Individual interviews and focus group interviews were conducted until data saturation was reached [22] and took place during NH and infant admission to the NICU, preventing recall bias.

### Conclusions

These data highlight the importance of NH for preterm infants. NH provides parents with a feeling of being a family and supports their self-efficacy. Parents do not request hands-on support for infant care but feel safe with nurse-provided security. Parents also outlined the need for a technological “bell cord” if NH is to be supported by telemedicine, which would consist of the following: possibility for video-conferencing, timely written email communication, and a knowledge base of information regarding infant nutrition and breastfeeding.

## Abbreviations

FCC: family-centered care
HCA: Hans Christian Andersen Children’s Hospital
HH: Hvidovre Hospital
NH: neonatal home care
NICU: neonatal intensive care unit
PD: participatory design
